# Baseline tumor gene expression signatures correlate with chemoimmunotherapy treatment responsiveness in canine B cell lymphoma

**DOI:** 10.1371/journal.pone.0290428

**Published:** 2023-08-25

**Authors:** Katherine Dittrich, Ümmügülsüm Yıldız-Altay, Fatima Qutab, Danny A. Kwong, Zechuan Rao, Sebastian A. Nievez-Lozano, Heather L. Gardner, Jillian M. Richmond, Cheryl A. London

**Affiliations:** 1 Cummings School of Veterinary Medicine at Tufts University, North Grafton, MA, United States of America; 2 UMass Chan Medical School, Worcester, MA, United States of America; Emory University, UNITED STATES

## Abstract

Pet dogs develop spontaneous diffuse large B cell lymphoma (DLBCL), and veterinary clinical trials have been employed to treat canine DLBCL and to inform clinical trials for their human companions. A challenge that remains is selection of treatment to improve outcomes. The dogs in this study were part of a larger clinical trial evaluating the use of combinations of doxorubicin chemotherapy, anti-CD20 monoclonal antibody, and one of three small molecule inhibitors: KPT-9274, TAK-981, or RV1001. We hypothesized that significant differential expression of genes (DEGs) in the tumors at baseline could help predict which dogs would respond better to each treatment based on the molecular pathways targeted by each drug. To this end, we evaluated gene expression in lymph node aspirates from 18 trial dogs using the NanoString nCounter Canine Immuno-oncology (IO) Panel. We defined good responders as those who relapsed after 90 days, and poor responders as those who relapsed prior to 90 days. We analyzed all dogs at baseline and compared poor responders to good responders, and found increased *CCND3* correlated with poor prognosis and increased *CD36* correlated with good prognosis, as is observed in humans. There was minimal DEG overlap between treatment arms, prompting separate analyses for each treatment cohort. Increased *CREBBP* and *CDKN1A* for KPT-9274, increased *TLR3* for TAK-981, and increased *PI3Kδ*, *AKT3*, and *PTEN*, and decreased *NRAS* for RV1001 were associated with better prognoses. Trends for selected candidate biomarker genes were confirmed via qPCR. Our findings emphasize the heterogeneity in DLBCL, similarities and differences between canine and human DLBCL, and ultimately identify biomarkers that may help guide the choice of chemoimmunotherapy treatment in dogs.

## Introduction

Diffuse large B cell lymphoma (DLBCL) is the most common form of lymphoma in canines. The majority of dogs experience remission with conventional multiagent chemotherapy, with median survival times of 10–12 months [1]. Yet many agents have high toxicities and unwanted side effects, and some pets remain recalcitrant to treatment. Thus, there has been recent interest in incorporating immunotherapy into existing chemotherapy treatment regimens in an attempt to improve survival times and reduce the amount of standard cytotoxic chemotherapy needed.

In human DLBCL, the immunotherapy drug rituximab, an anti-CD20 antibody, has greatly improved survival times [2]. The tumor microenvironment (TME), made up of tumor-adjacent stromal and immune cells, also influences patient outcome in human DLBCL. For example, patients with higher levels of tumor-infiltrating CD4+ T cells have improved survival [3], and patients with increased immune checkpoint positive T cells have poorer outcomes [4]. Likewise, in a study of non-neoplastic lymphocytes in the lymph nodes of dogs with DLBCL, a greater percentage of tumor-infiltrating T cells as assessed with flow cytometry was correlated with a longer time to progression [5]. Despite these advances in our understanding of the TME, the mechanisms underlying its impact on prognosis and response to treatment in DLBCL remain undefined. Thus, there is a gap in our understanding of how the immune landscape influences response to such treatment protocols. Understanding the gene expression profile of the DLBCL TME and neoplastic cells themselves will improve our understanding of how to use immunotherapy in DLBCL in both human and veterinary medicine.

Several human DLBCL studies have created gene signature panels incorporating tumor microenvironment-related genes that predict survival [6–8]. For example, Merdan et al. performed RNA-sequencing on tumor samples and gene expression analysis using edgeR package to create gene expression-based scores that were associated with long-term survival [6]. Similarly, Pan et al. used the Gene Expression Omnibus database of patients with DLBCL to identify a seven-gene signature of TME-related genes that held prognostic value [7]. However, not all immune signatures studied have found significant biologic correlates, supporting the notion that a better understanding of the immune micro environment is necessary to validate prognostic biomarkers associated with response to therapy [4]. Finally, the variability in predictive potential of gene expression panels also suggests that the genetic context of these gene expression patterns are important determinates of response both in the immune system and the tumor itself [8, 9].

Attempts to use gene expression analysis as a predictive model in canine DLBCL are comparatively few and have not been prospectively validated. Most of these approaches aim to assess canine DLBCL within the same molecular classification scheme used in human DLBCL. For example, gene expression profiling categorized canine DLBCL in activated B-cell (ABC)-like and germinal center B-cell (GCB)-like categories, reminiscent of the ABC and GCB molecular subtypes in human DLBCL, including NF-κB pathway dysregulation [10]. Additional efforts to re-analyze this dataset identified additional genes and pathways associated with canine DLBCL, including genes associated with the PI3K/AKT pathway (*PTEN*, *PIK3CG*, *PLCB4* and *INPP4B*), which holds prognostic value in human DLBCL [11]. Another group evaluated a set of 36 genes prognostic in human DLBCL on two canine B cell lymphoma microarray datasets, then verified their findings using qPCR on lymph nodes of 60 dogs with B cell lymphoma. however only one gene (*CCND1*) was found to be prognostic in dogs on multivariate analysis, suggesting that the genetic landscape does not directly correlate amongst different species [12]. Curran at el. evaluated expression of MYC and BCL2 in canine DLBCL via immunohistochemistry [13]. Though lymph node samples in that study showed expressed of both proteins, neither was associated with prognosis, underscoring the importance of functional validation of proposed genes [13]. Several other techniques have been used to assigned prognostic value to the TME in canine B cell lymphoma, including flow cytometry, cytokine profiling, and evaluation of checkpoint molecules [5, 14–16]. However, flow cytometry is limited by the antibodies that are available, and many of these studies are based on retrospective sample collection. Therefore, there is still much to be learned, especially in the context of the role of the tumor microenvironment in response to specific chemo-immunotherapies.

Our study sought to better characterize gene expression in canine DLBCL and its associated immune landscape by evaluating gene expression in lymph node aspirates from affected dogs using the NanoString nCounter® Canine Immuno-oncology Panel (canine IO panel). We hypothesized that use of this technology would lead to identification of genes that would accurately distinguish dogs that respond well to chemo-immunotherapy (e.g. dogs that experienced disease progression after 90 days vs those who experienced disease progression prior to 90 days). Dogs enrolled in this trial received doxorubicin chemotherapy, anti-CD20 monoclonal antibody, and one of three small molecule inhibitors: KPT-9274, TAK-981, or RV1001. We chose these 3 immunomodulatory therapies due to their proposed mechanisms of action in enhancing immune activation to fight tumors. We aimed to determine whether the baseline lymph node aspirate gene expression profiles could predict treatment responsiveness and outcome with these combinations of chemo-immunotherapy, and present here potential candidate biomarkers that align with the mechanism of action of the small molecule inhibitors.

## Results

### Patient demographics and trial outcomes

Eighteen dogs with DLBCL were included in this study with owner consent and IACUC approval. Baseline lymph node aspirates were available from all 18 dogs, and progressive disease (PD) lymph node aspirates were available in 15 dogs. The reasons for lack of a PD sample in three dogs are as follows: one dog developed T cell lymphoma while in remission for DLBCL, one dog moved away while still in remission, and one dog was removed from the study prior to PD due to pyelonephritis. Cohort demographics are detailed in S1 Table in S1 File.

**Fig 1:**
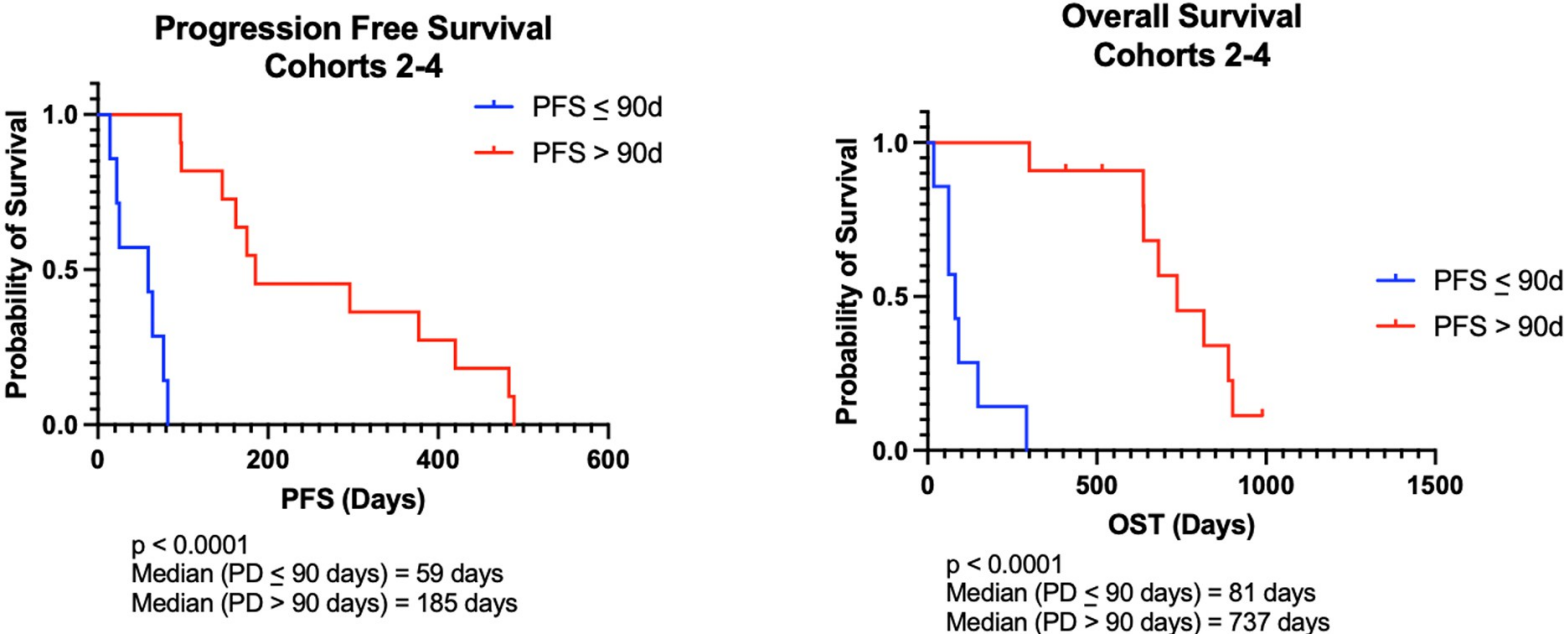
Survival outcomes are improved in canine DLBCL patients receiving chemoimmunotherapy who progress after 90 days. (A) Kaplan-Meier analysis for progression free survival (PFS) and (B) overall survival time (OST) in dogs who progressed before and after 90 days. Both PFS and OST were statistically significant between the groups (p<0.0001.) Tick marks indicate censored patients.

For all dogs, median progression free survival (PFS) was 122 days (range, 14-489d) and median survival time (MST) was 462 days (range, 18–989 days). For dogs whose lymphoma progressed prior to 90 days (n = 7), median PFS was 59 days (range, 14–82 days) and MST was 81 days (range, 18–292 days). For dogs whose lymphoma progressed after 90 days (n = 11), median PFS was 185 days (range, 97–489 days) and MST was 737 days (range, 300–989 days). The differences in both PFS and MST between the two groups of responders were statistically significant with p<0.0001 (Fig 1). The 90-day cutoff was determined from clinical patterns of early failure associated with standard CHOP-based chemotherapy. Specifically, dogs that fail therapy within the first cycle (e.g. within 3–4 weeks of starting chemotherapy) tend to have shorter remissions. This cutoff also represents a natural split in outcomes associated with our study population.

Comparisons of patient demographics and variables between the two groups of responders are depicted in Table 1. Average age was similar in the two groups. The group that progressed after 90 days had a greater number of mixed breed dogs. Castrated males and spayed females were more common than intact dogs in both groups. Most dogs in each group were stage IIIa at diagnosis. Dogs were fairly evenly distributed amongst the trial arms in each group.

**Table 1:** Comparison of characteristics between patients whose disease progressed prior to and after 90 days.

Characteristics	PD<90 Days	PD >90 Days
**Number of patients**	7	11
**Mean age (years) at diagnosis (range)**	7.6 (5–10)	7.0 (3–11)
**PFS (range)**	59 (14–82)	185 (97–489)
**MST (range)**	81 (18–292)	737 (300–989)
**Breed**	N (%)	N (%)
Mixed breed	1 (14)	7 (64)
Pitbull	1 (14)	2 (18)
Mastiff	1 (14)	0
Yorkshire Terrier	1 (14)	0
Bulldog	1 (14)	0
Cattle dog	1 (14)	0
Scottish Terrier	1 (14)	0
German Shepherd	0	1 (9)
Pug		1 (9)
**Gender**	N (%)	N (%)
Castrated male	3 (43)	4 (36)
Spayed female	3 (43)	6 (55)
Intact male	1 (14)	0
Intact female	0	1 (9)
**Stage**	N (%)	N (%)
IIIa	4 (57)	6 (55)
IIIb	0	1 (9)
IVa	2 (29)	3 (27)
IVb	1 (14)	1 (9)
Va	0	0
**Trial arm**	N (%)	N (%)
KPT-9274	3 (43)	4 (36)
TAK-981	2 (29)	3 (27)
RV1001	2 (29)	4 (36)

PD, progressive disease; PFS, progression free survival; MST, median survival time. Age is represented in years as a mean (range). PFS and MST are represented in days as a median (range). Breed, gender, stage, and trial arm are shown as number (percent).

#### Differential gene expression is heterogeneous when all treatment arms are analyzed together

**Fig 2:**
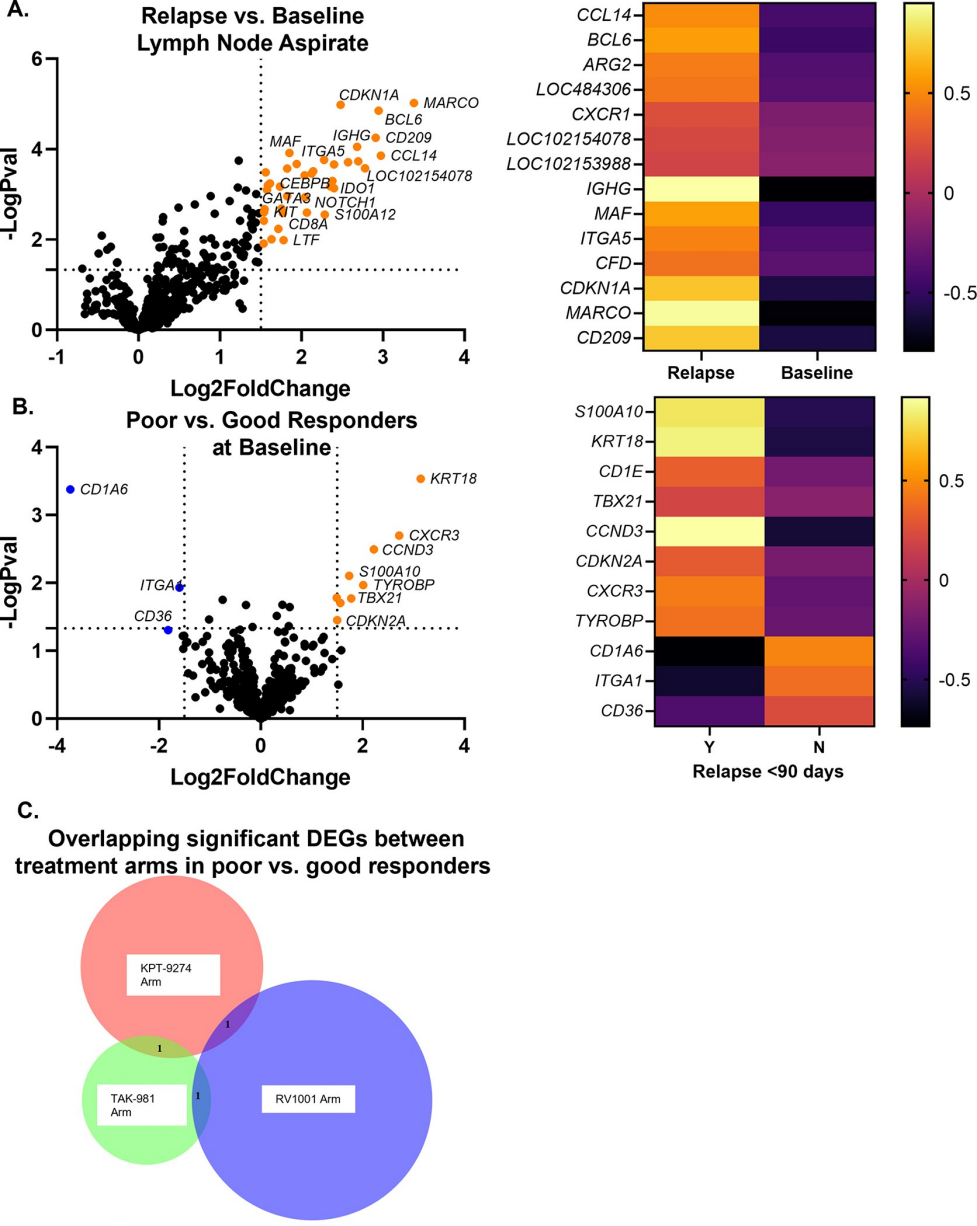
Differential gene expression for all dogs. (A) Differentially expressed genes at time of PD as compared to baseline. Colored dots in the volcano plot represent genes that met significance criteria (n = 14) of log2fold change < or > 1.5 and padj<0.01. The heatmap was made with the same significance criteria. (B) Differentially expressed genes in dogs who progressed prior to 90 days as compared to dogs who progressed after 90 days. Colored dots in the volcano plot represent genes that met significance criteria (n = 11) of log2fold change < or > 1.5 and p<0.05. The heatmap was made using the same significance criteria. (C) When each arm was analyzed separately using p<0.05, regardless of log2fold change, there were zero overlapping significant differentially expressed genes between all three arms in dogs who progressed prior to 90 days and those who progressed after 90 days.

Differentially expressed genes (DEGs) were first analyzed between all dogs at time of PD as compared to baseline. There were 14 significantly upregulated genes at time of PD as compared to baseline, and no genes were significantly downregulated (Fig 2A, log2fold change < or > 1.5 and padj<0.01.) The upregulated genes that met this significance threshold were *MARCO*, *CCL14*, *BCL6*, *CD209*, *LOC102154078*, *LOC102153988*, *IGHG*, *LOC484306*, *CDKN1A*, *CXCR1*, *ITGA5*, *ARG2*, *MAF*, and *CFD* (S2 Table in S1 File). The top three gene sets as determined by NanoString analysis, which were all upregulated at time of PD, were the myeloid compartment, senescence, and metabolic stress (S3 Table in S1 File).

All baseline samples were then analyzed, comparing differentially expressed genes in dogs who progressed prior to 90 days (from now on referred to as poor responders) as compared to dogs who progressed after 90 days (from now on referred to as good responders.) There were 11 significantly differentially expressed genes (Fig 2B, log2fold change < or > 1.5 and p<0.05.) Eight genes were upregulated in poor responders at baseline: *KRT 18*, *CXCR3*, *CCND3*, *TYROBP*, *TBX21*, *S100A10*, *CD1E*, and *CDKN2A*. Three genes were downregulated in poor responders at baseline: *CD1A6*, *CD36*, and *ITGA1* (S4 Table in S1 File). Only one gene set, NK cell functions, had a significance score above 1.5; this term was upregulated in poor responders (S5 Table in S1 File).

Each trial arm was analyzed separately, comparing poor responders to good responders within each group. When using p<0.05, regardless of log2fold change, there were no genes that overlapped between all three arms (Fig 2C). There was one gene that overlapped between the KPT-9247 arm and the TAK-981 arm (*IFNGR1*), one gene that overlapped between the KPT-9274 arm and the RV1001 arm, (*FCER2*), and one gene that overlapped between the TAK-981 arm and the RV1001 arm (*KRT18*.) This finding prompted more detailed analysis of each treatment arm individually.

#### Candidate biomarker gene expression in KPT-9274 arm correlates with drug mechanism targeting PAK4-CREB-CDKN1A

**Fig 3:**
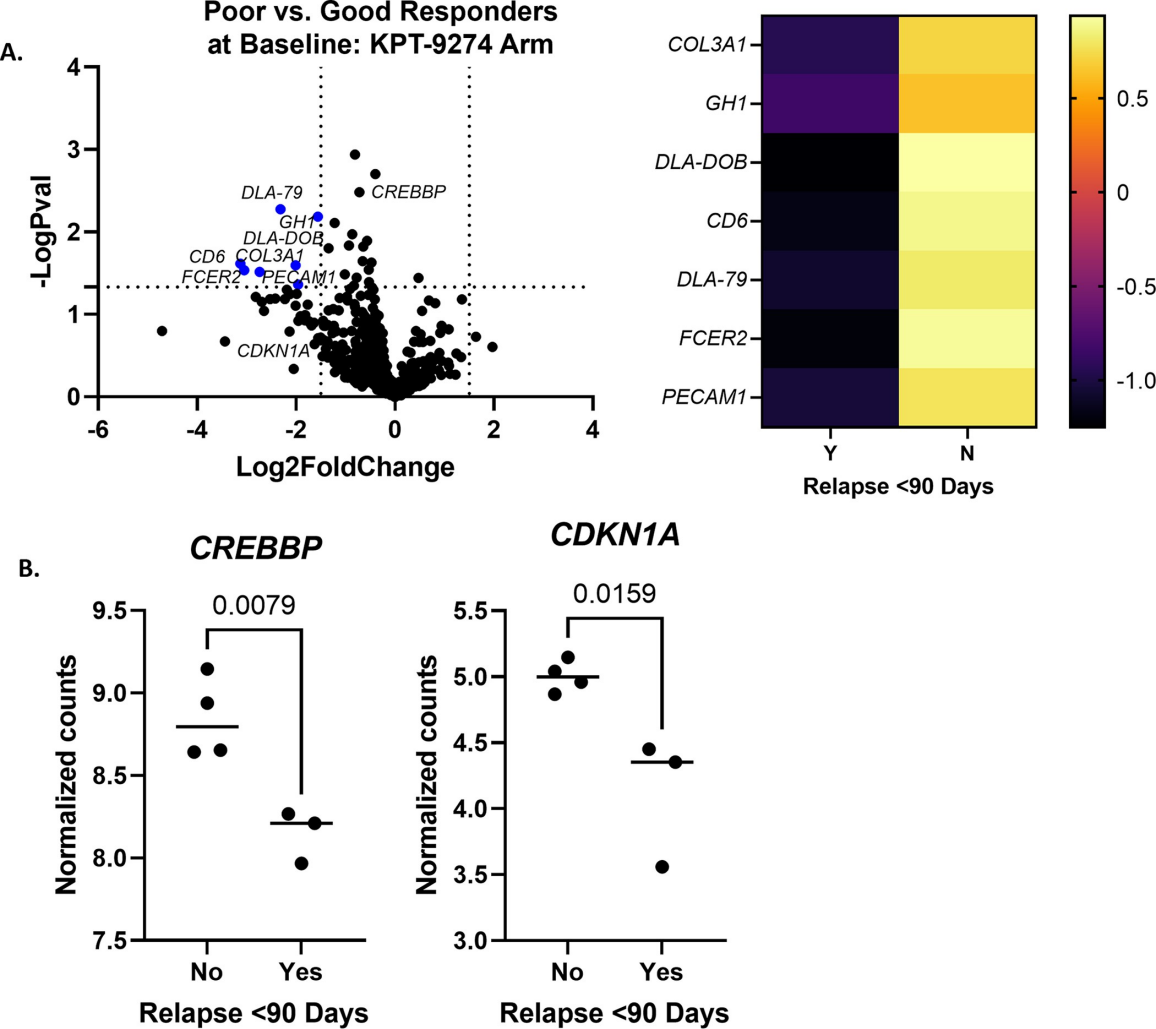
KPT-9274 arm differential gene expression between poor and good responders at baseline. (A) Differentially expressed genes in poor responders as compared to good responders (n = 3 poor and 4 good responders; log2fold change < or > 1.5 and p<0.05.) A heatmap was generated with the same significance criteria. (B) When analyzed individually, normalized gene counts for *CREBBP* were significantly higher in good responders using an unpaired t-test (p = 0.0079). Normalized gene counts for *CDKN1A* were also significantly higher in good responders using an unpaired t-test (p = 0.0159).

For dogs in the KPT-9274 arm (see Methods section), there were seven differentially expressed genes between poor and good responders at baseline (n = 3 poor and 4 good responders; Fig 3A, log2fold change < or > 1.5 and p<0.05.) All seven genes were downregulated in poor responders at baseline: *CD6*, *FCER2*, *COL3A1*, *DLA-79*, *DLA-DOB*, *PECAM1*, and *GH1* (S6 Table in S1 File). Four gene sets had a significance score above 1.5 and all were downregulated in poor responders at baseline; these were antigen processing, Notch signaling, Wnt signaling, and epigenetic regulation (S7 Table in S1 File).

Individual gene expression analysis was performed for *CREBBP*, due to its relationship to KPT-9274’s mechanism of action, encoding for a binding protein of CREB, which acts downstream of PAK4 [17]. It was found to be significantly higher in good responders (Fig 3B, p = 0.0079). The expression of *CDKN1A* was also analyzed individually in dogs in the KPT-9274 arm at baseline, due to its potential relationship to the mechanism of KPT-9274 as being downregulated by PAK4 [18, 19]. *CDKN1A* expression was significantly higher in good responders (Fig 3C, p = 0.0159.) Other genes potentially related to the mechanism of KPT-9274 were either not available in the panel (*PAK4*, *NAMPT*, *CTNNB1*, *CDC42*, *BAD*, *MYLK*) or were not significant between the groups of responders (*RAF-1*, *GSK3B*).

#### Candidate biomarker gene expression in TAK-981 arm is related to IFN signaling via TLR3

**Fig 4:**
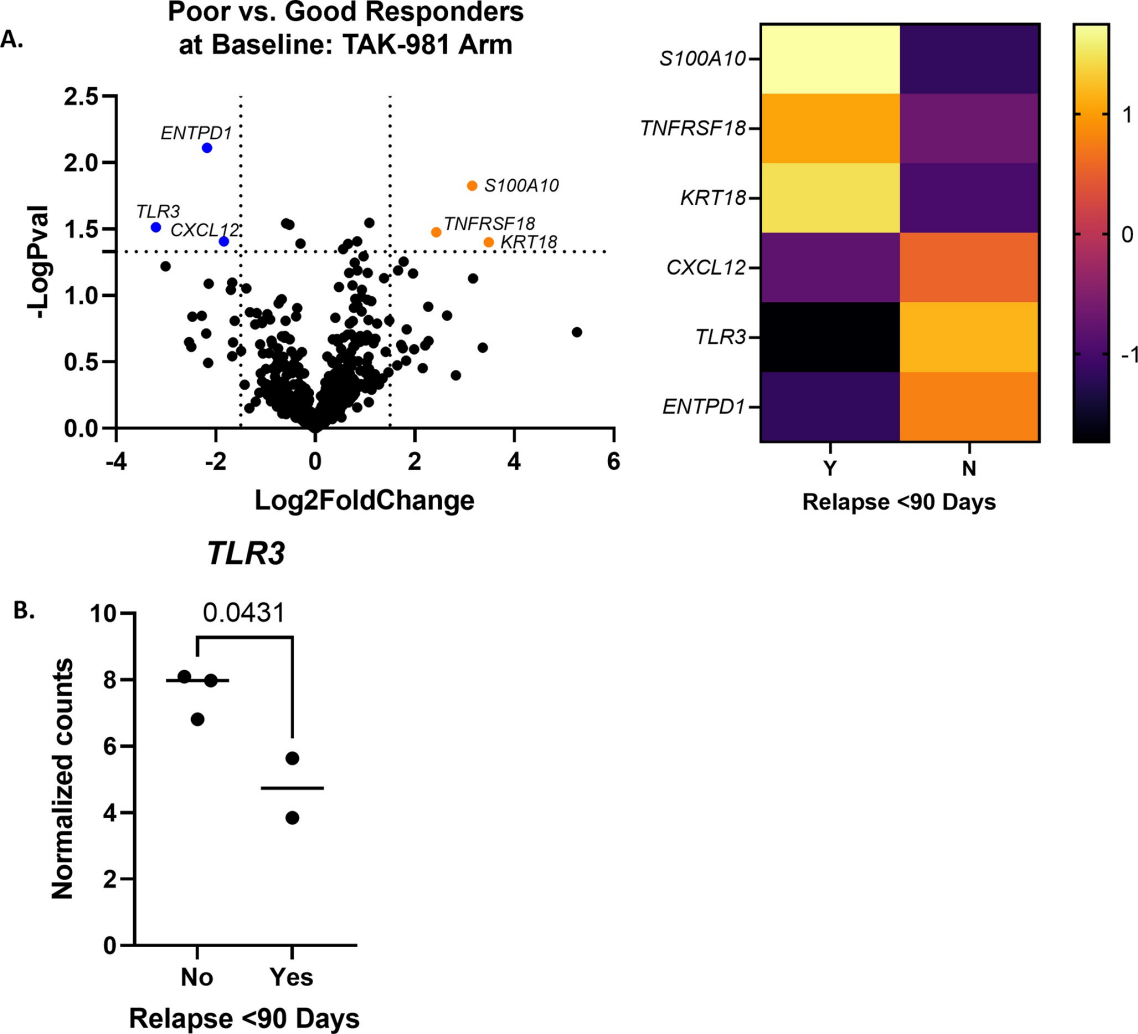
TAK-981 arm differential gene expression between poor and good responders at baseline. (A) Differentially expressed genes in poor responders as compared to good responders (n = 2 poor and 3 good responders; log2fold change < or > 1.5 and p<0.05.) A heatmap was made with the same significance criteria. (B) Normalized gene counts for *TLR3* were significantly higher at baseline in good responders (p = 0.0431 with unpaired t test).

For dogs in the TAK-981 arm, there were six differentially expressed genes between poor and good responders at baseline (n = 2 poor and 3 good responders; Fig 4A, log2fold change < or > 1.5 and p<0.05.) Three genes were upregulated in the poor responders (*KRT18*, *S100A10*, and *TNFRSF18*), and three genes were downregulated in the poor responders (*TLR3*, *ENTPD1*, *CXCL12*) (S8 Table in S1 File). No gene sets reached a significance above 1.5 (S9 Table in S1 File).

The expression of *TLR3* was analyzed individually in dogs in the TAK-981 arm at baseline, due to its relationship to the mechanism of action of TAK-981, which upregulates genes associated with the type I interferon response [20]. *TLR3* expression was significantly upregulated in good responders at baseline as compared to poor responders (Fig 4B, p = 0.0431 with unpaired t-test.) A select number of other genes related to the IFN Type I pathway were chosen for individual analysis based on a significant p value in Rosalind analysis. These included *IFNAR1* and *IFNAR2*, and were not found to be significant between the two groups of responders on either an unpaired t test or Mann Whitney test.

#### Gene expression in RV1001 arm correlates with PI3K pathway

**Fig 5:**
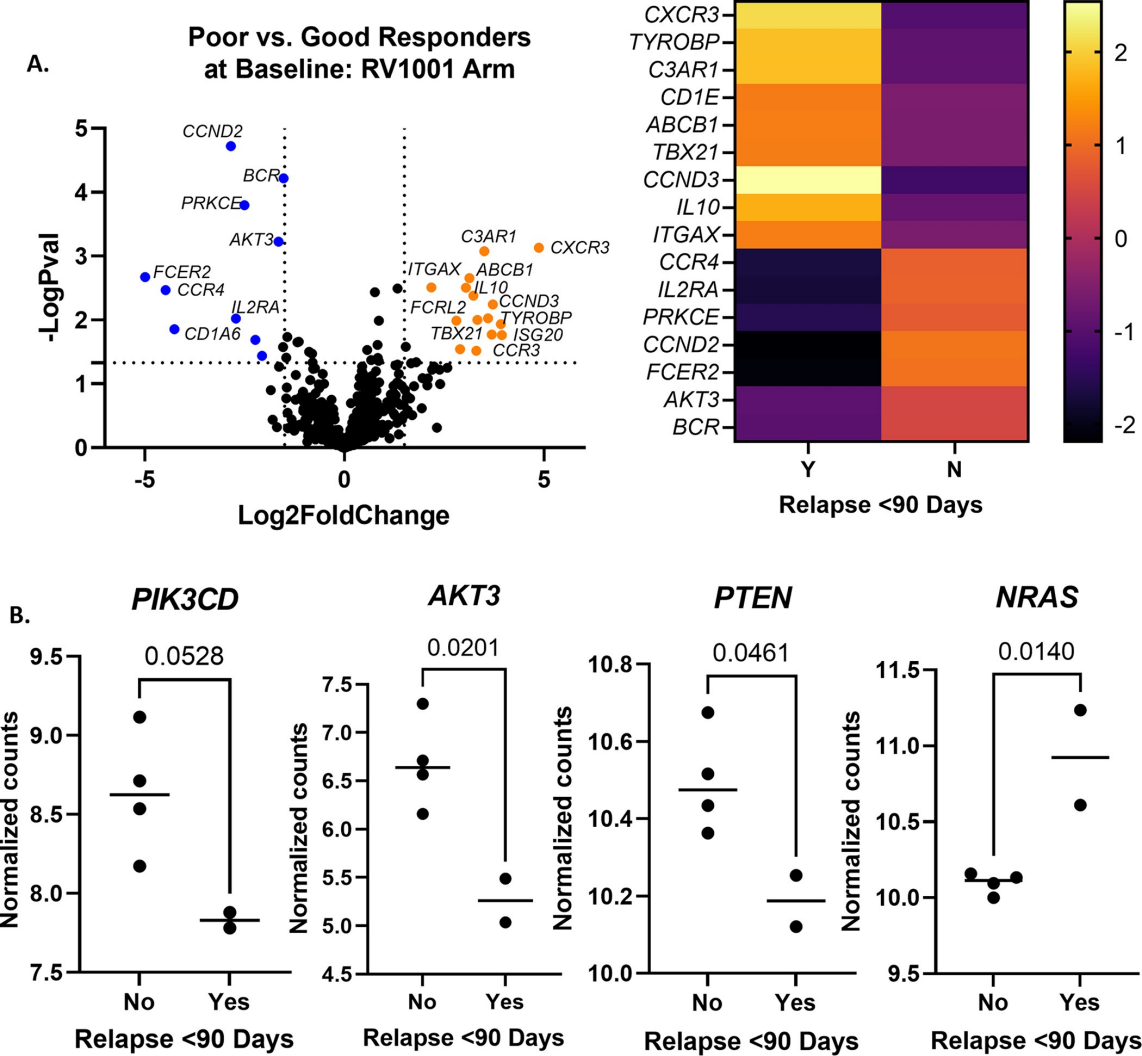
RV1001 arm differential gene expression between poor and good responders at baseline. (A) Differentially expressed genes in poor responders as compared to good responders (n = 2 poor and 4 good responders; log2fold change < or > 1.5 and p<0.01.) A heatmap was constructed using the same significance criteria. (B) Normalized gene counts for *PIK3CD* were higher in good responders, and approached significance (p = 0.0528.) Normalized gene counts for *AKT3* and *PTEN* were significantly upregulated in good responders (p = 0.0201 and 0.0461, respectively.) Counts for *NRAS* were significantly upregulated in poor responders (p = 0.01.) Comparisons were made using unpaired t tests.

Poor responders in the RV1001 arm had an excess of differentially expressed genes, prompting more stringent significance criteria. There were 16 differentially expressed genes between poor responders and good responders (n = 2 poor and 4 good responders; Fig 5A, log2fold change < or > 1.5, p<0.01.) Nine genes were upregulated in poor responders at baseline (*CXCR3*, *CCND3*, *TYROBP*, *C3AR1*, *TBX21*, *CD1E*, *ABCB1*, *IL10*, *ITGAX*) and seven genes were downregulated in poor responders at baseline (*FCER2*, *CCR4*, *CCND2*, *IL2RA*, *PRKCE*, *AKT3*, *BCR*) (S10 Table in S1 File). There were five gene sets that reached significance above 1.5, all of which were upregulated in poor responders at baseline. These were cell proliferation, TGF-beta signaling, interleukins, Wnt signaling, and NK cell functions (S11 Table in S1 File).

Several genes deemed related to the mechanism of RV001 were analyzed individually in dogs in this arm at baseline (Fig 5B). *PIK3CD*, which encodes for the primary target of RV1001, was upregulated in good responders as compared to poor responders. This difference neared significance (p = 0.0528.) *AKT3*, a downstream effector of PIK3CD, was also higher in good responders as compared to poor responders. This difference was significant (p = 0.02.) *PTEN*, which negatively regulates the PI3K pathway, was significantly upregulated in good responders (p = 0.461.) NRAS, which acts horizontally to and can activate PI3K pathways [21], was significantly upregulated in poor responders (p = 0.01.) Other genes in the pathway that were analyzed individually and were not significant were *AKT1* and *MTOR*.

### Confirmation of candidate biomarkers using real time PCR

**Fig 6:**
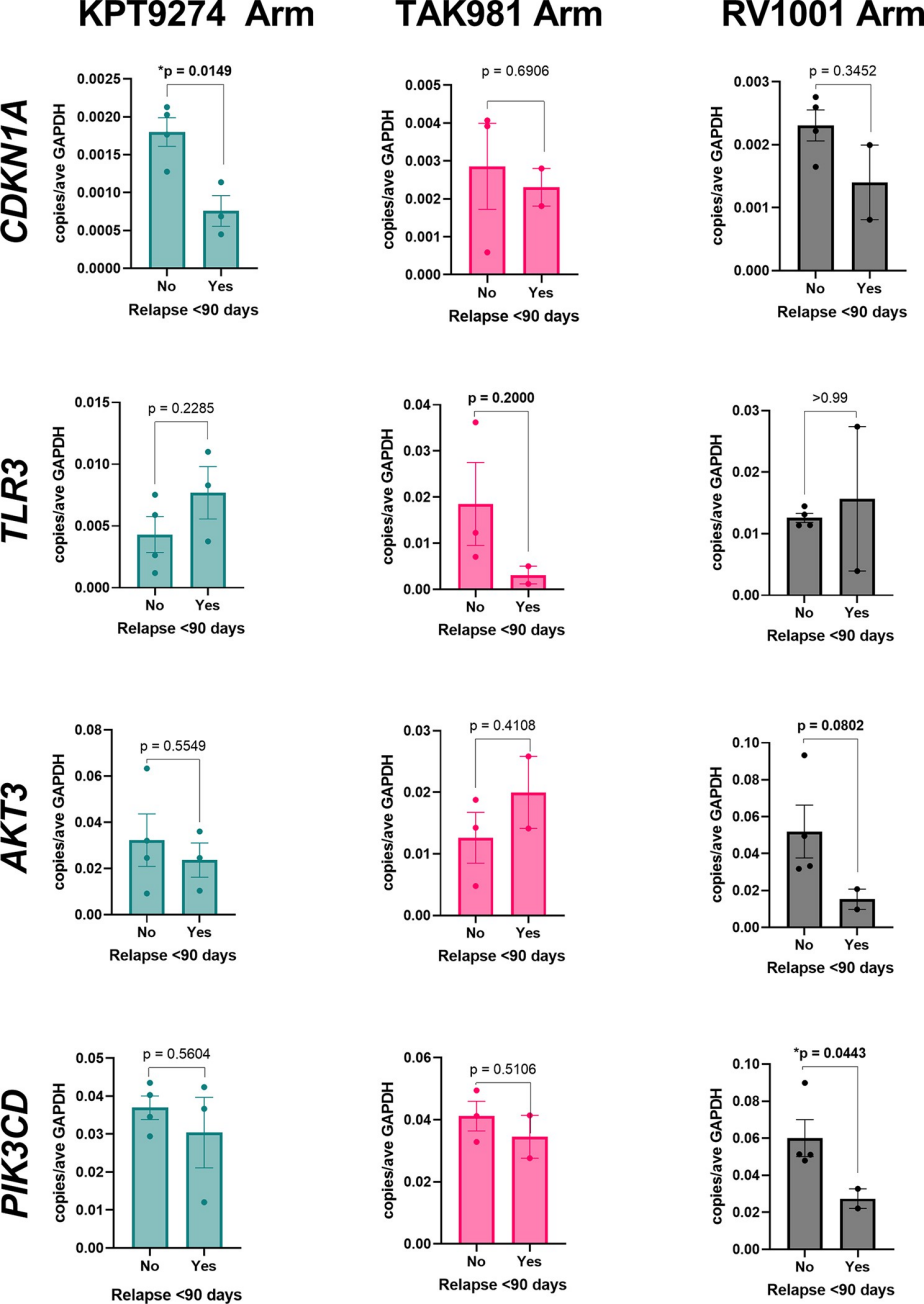
Confirmation of select differential gene expression using qPCR. Select genes were analyzed for differential expression between good and poor responders (relapse before or after 90 days) in each treatment arm using qPCR. *CDKN1A* was significantly upregulated in good responders in the KPT-9274 arm (p = 0.0149.) *TLR3* was upregulated in good responders in the TAK-981 arm, but was not significant (p = 0.2000). *AKT3* was upregulated in good responders in the RV1001 arm, and neared significance (p = 0.0802). *PIK3CD* was upregulated in good responders in the RV1001 arm, and was significant (p = 0.0443). Comparisons were made using unpaired t-tests.

A select number of genes deemed significant via Nanostring’s nCounter® methods were analyzed via qPCR to confirm differential expression (Fig 6). *CKDN1A*, which was significantly upregulated in good responders who received KPT-9274 via nCounter® analysis, was also significantly upregulated in these dogs via qPCR. By comparison, *CDKN1A* expression was not significant between good and poor responders in the other treatment arms. *TLR3*, which was significantly upregulated in good responders who received TAK981 via nCounter® analysis, was overexpressed in this same group of dogs via PCR, though did not reach significance. For dogs who received RV1001, *AKT3* and *PIK3CD* were confirmed to be overexpressed in good responders via qPCR (with significance for *PIK3CD* and nearing significance for *AKT3*). Differential expression for these two genes was not significant in the other two treatment arms.

## Discussion

This study sought to characterize gene expression within the tumor microenvironment and neoplastic lymphocytes of dogs with DLBCL, and to determine if differential expression of these genes could discriminate exceptional responders treated with specific chemoimmunotherapy treatment regimens. Similar NanoString panels have been used successfully in human DLBCL [4, 22–24], prompting the use of a comparable canine panel for this study.

We first evaluated dogs in all treatment arms as a group, comparing differential gene expression at time of PD as compared to baseline. All genes meeting the threshold for significance were upregulated at time of time of PD, a few of which merit further discussion. The most significantly upregulated gene at time of PD was *MARCO*, which is associated with M2 tumor associated macrophages (TAMs) and has been shown to be upregulated in human DLBCL TAMs [25]. TAMs promote tumor progression through immunosuppression and expression of checkpoint molecules, among other mechanisms, and are a therapeutic target of interest in relapsed/refractory human DLBCL [26]. They have also been associated with shorter survival times in human DLBCL [27]. The upregulated expression of *MARCO* at time of PD in our patients may represent a greater infiltration of immunosuppressive and tumor promoting TAMs at the time of PD.

Another significantly upregulated gene at time of PD was *BCL6*, which is required for the proliferation of germinal center B cells, and is expressed in most human DLBCLs, where expression is higher in the germinal center B-cell (GCB) subtype than in activated B-cell (ABC) subtype [28]. Enrichment in *BCL6* expression has not been identified in relapsed human DLBCL as compared to baseline [29], in contrast to our cohort of dogs. Sato et al. evaluated *BCL6* expression in a group of dogs with untreated DLBCL, and did not find a correlation with prognosis, unlike in humans. Additionally, mRNA expression was lower than in their control dogs. All cases evaluated by IHC in that study were negative for BCL6 protein expression [30], and BCL6 was rarely detected via IHC by dogs with DLBCL in another study [10]. The previously mentioned study by Zamani et al. failed to identify *BCL6* as a hub gene [11]. Further evaluation is required to fully elucidate the significance of upregulation in *BCL6* in our dogs at time of PD, but in light of these previous studies, it may be that *BCL6* only becomes significant at time of relapse or progressive disease in dogs.

It is also worth noting that *CDKN1A*, which encodes for p21, a negative regulator of cell cycle progression and downstream effector of p53 [31], was upregulated at time of PD. TP53 mutations are higher in relapsed and refractory human DLBCL as compared to primary cases [29]. Therefore, it is surprising that *CDKN1A* is upregulated at time of PD in our patients, though it’s possible that a mutation in upstream TP53 could lead to increased expression of *CDKN1A*. It is also surprising because a previous study found that *CDKN1A* is downregulated in canine DLBCL [11]. An alternative theory is that *CDNK1A* was upregulated at time of PD in our patients in response to DNA damage from chemotherapy agents [32]. Overall, there were many genes that were upregulated at the time of PD in our patients, but a clear pattern did not emerge, and the data is difficult to interpret due to the variations in time of PD as compared to completion of chemotherapy, in addition to the variations in treatment protocols and heterogeneity of study subjects. However, TAMs and BCL6 may represent a therapeutic target in dogs with progressive DLBCL.

Next, we analyzed dogs from all three treatment arms as a group to see if there were differentially expressed genes between poor responders and good responders. Genes that stood out as having potential prognostic significance based on the literature for human and canine DLBCL were *TBX21*, *CDKN2A*, *CCND3*, and *CD36*. *TBX21*, which encodes for transcription factor T-bet, was significantly upregulated in poor responders. In humans, T-bet expression via immunostaining was identified in precursor B-cell lymphoblastic leukemia/lymphoma, B-cell chronic lymphocytic leukemia, and marginal zone lymphoma, but not in DLBCL [33]. Thus, the poor responders with upregulated *TBX21* expression may have had a more aggressive phenotype that was not captured on our flow cytometry panel. *CDKN2A*, a cyclin-dependent kinase inhibitor, was also significantly upregulated in poor responders. Interestingly, this is in direct contrast to reports of human DLBCL, where *CDKN2A* deletion is associated with poor survival and correlates with decreased gene expression [34]. This finding may represent a distinct difference between human and canine DLBCL, though would need to be validated in a larger set of dogs. *CCND3*, which encodes for cyclin D3, was significantly upregulated in poor responders, and overexpression via IHC has been correlated with a poor prognosis in human DLBCL [35]. Ulve et al. found a fusion between *IGK* and *CCND3* in one canine case of DLBCL, resulting in overexpression of *CCND3*, though its effect on prognosis was not reported [36]. Finally, *CD36* was downregulated in the poor responders; in human DLBCL, CD36 overexpression via IHC was shown to improve prognosis in patients receiving R-CHOP [37]. Thus, *CD36* expression may represent a shared positive prognostic indicator between humans and dogs.

KPT-9274, a NAMPT/PAK4 inhibitor, has shown efficacy in dogs when used with doxorubicin [38]. In the KPT-9274 arm, one gene of note that was downregulated in poor responders is *DLA-DOB*, which encodes for a class II MHC protein. A few studies in dogs with B-cell lymphoma have demonstrated that low levels of class II MHC carry a poorer prognosis [16, 39], in line with our findings in this arm, though in a different study it was not found to be prognostic for DLBCL [40]. Several of the significantly downregulated gene sets in poor responders are also worth mentioning. Notch and Wnt signaling were downregulated in poor responders. The dogs in this arm received KPT-9274, which is a dual inhibitor of PAK4 and NAMPT; PAK4 has been implicated as being involved in both Wnt and Notch pathways [17, 41]. Thus, we suspect that the good responders may have had upregulation in PAK4, Wnt, and Notch pathways, which allowed them to respond better to PAK4 inhibition with KPT-9274. Indeed, KPT-9274 was shown to inhibit Notch signaling in human rhabdomyosarcoma tumors [41]. Although *PAK4* and *NAMPT* were not available in our gene expression panel for analysis, we analyzed the expression of *CREBBP*, which encodes for a binding protein of CREB that is activated by PAK4 [17]. Gene expression of *CREBBP* was significantly higher in good responders at baseline (Fig 3C), and since it acts downstream of PAK4, this may again indicate that KPT-9274 targeting of PAK4 was more effective in this group of dogs. On the other hand, Scialdone et al. found that in DLBCL cell lines depleted of CREB binding protein, the response to anti-CD20 antibody was impaired [42]. Since dogs in this study also received anti-CD20 antibody, the difference in expression of *CREBBP* between the two groups of responders could also be explained by this finding. We also looked at *CDKN1A*, which encodes for p21, and is thought to be downregulated by PAK4 [19]. This gene was significantly higher in good responders at baseline (Fig 3C), which is surprising, if it is indeed downregulated by PAK4. Overall, the findings from the KPT-9274 arm suggest that increased expression of *CREBBP*, *CDKN1A*, and genes related to Wnt and Notch signaling may predict better response to KPT-9274.

TAK-981 is a SUMO-activating enzyme inhibitor and induces genes associated with Type I interferon responses [20]. In the TAK-981 arm, genes related to interferon signaling were examined, since this drug upregulates genes associated with the Interferon Type I response [20]. Although overall gene set analysis of interferon signaling as determined by NanoString was not deemed significant (significance score 1.0353), when genes were analyzed individually, *TLR3* was significantly higher in good responders at baseline. TLR3 is known to induce genes associated with Type I Interferons [43], so this effect may have been enhanced even further by TAK-981 in these dogs. However, the fact that other genes associated with the IFN-I pathway were not significant, and that no gene sets reached a global significance score above 1.3, suggests that the gene signature in this group of dogs was extremely heterogenous. There may also be other, yet unknown pathways targeted by TAK-981, though dogs with higher expression of *TLR3* may respond better to the drug.

RV1001 is a PI3Kδ inhibitor, a pathway known to be enriched in human and canine DLBCL [11] and showed an objective response rate of 86% in a phase II study in dogs with DLBCL [44]. Of the upregulated genes in poor responders in the RV1001 arm, *ABCB1* is not surprising. Doxorubicin, which was received by all dogs in this study, is a known substrate of ABCB1, the drug efflux pump also known as p-glycoprotein [45]. Drug resistance to doxorubicin has been associated with increased p-glycoprotein in canine B-cell leukemia cell lines, and increased ABCB1 expression was associated with acquired (but not intrinsic) drug resistance to doxorubicin in dogs with B cell lymphoma in another study [46, 47]. In that study, pre-treatment expression of ABCB1 was not predictive of outcome, unlike in our small group of dogs. Another gene of interest that was significantly upregulated in poor responders in this arm is *IL10*. In human DLBCL, patients with higher serum IL-10 had a shorter event-free survival [48], and serum IL-10 is increased in dogs with DLBCL as compared to controls [14, 49], although less is known about its impact on prognosis in dogs. Other upregulated genes of interest in the poor responders, namely *CCND3* and *TBX21*, were discussed above with all treatment groups combined.

RV1001 inhibits PI3Kδ, so we examined genes related to the PI3K/Akt pathway to see if they influenced responsiveness to the drug. *PIK3CD* is the gene that encodes for the primary target of RV1001 (PI3Kδ) and was increased in good responders at baseline with approaching significance. Likewise, *AKT3* was significantly increased in good responders; *AKT3* encodes for an isoform of one of the three Akt proteins, which are downstream effectors of PI3K [50], and phosphorylated AKT has been shown to be inhibited by RV1001 [44]. These findings suggest that dogs with upregulation in the PIK3/Akt pathway responded better to RV1001, likely because neoplastic cells were relying on pathways targeted by the drug. *PTEN*, whose product inhibits the PI3K pathway, was higher in good responders, which is puzzling if we think that the pathway was upregulated in this group, though PTEN has roles in other effector pathways as well [51]. Additionally, the gene could have been mutated in this group of dogs, which would not have been captured in our analysis and could affect expression data. Interestingly, the gene encoding for NRAS, which operates horizontally to but can activate PI3K, was upregulated in poor responders. RAS has a variety of other effector pathways (such as RAF-MEK-ERK), which may explain its role in conveying a poorer prognosis [21]. In sum, increased expression of *PIK3CD*, *AKT3*, and *PTEN*, and downregulation of *NRAS*, may predict which dogs will respond better to RV1001.

PCR confirmed the gene expression trends for several of the genes in this study, including *CKDN1A*, *TLR3*, *AKT3*, and *PIK3CD*. These findings via PCR validate our methods of gene expression analysis via Nanostring’s nCounter®. Though several of them met significance criteria, we suspect that those that did not were due to low case numbers, and the overall trends were still valid.

This study has several limitations. One important caveat is that we did not distinguish the neoplastic cells from tumor-infiltrating immune cells and stromal cells in our gene expression analysis. While we surmise that many of the differentially expressed genes, such as the ones involved in the PI3K/Akt pathway, were representative of the neoplastic cells, we cannot rule out contribution from other immune cells. Other genes mentioned in this study, such as macrophage-specific *MARCO*, surely represent immune cells in the tumor microenvironment. A future study could repeat the analysis using single cell sequencing or digital spatial analysis to separate neoplastic cells from other cell types. Additionally, dogs in each arm received different treatment combinations, which made analyzing them all in one group difficult. This was mitigated by analyzing gene expression within each treatment arm, though that approach resulted in fewer sample numbers for each analysis. Findings would need to be corroborated in a larger cohort of dogs. Also, we focused on the mechanisms of the small molecular inhibitors in each treatment arm, but outcome was likely also related to their response to doxorubicin and anti-CD20 antibody. We also acknowledge that there are many steps in between gene expression and the protein level, so future studies could use IHC to corroborate with protein expression. Finally, the NanoString nCounter® Canine IO panel has a limited selection of 800 genes, and there are likely many more differentially expressed genes that contributed to the varying responses of these dogs.

In conclusion, this study evaluated differential gene expression in a group of dogs with DLBCL receiving a variety of chemoimmunotherapy treatments. Our findings emphasized the tremendous heterogeneity in the tumor and surrounding microenvironment, which indicates the need for more tailored treatment regimens in dogs with DLBCL. We uncovered a few prognostic genes that overlapped with those reported in humans (*CCND3* as a poor prognostic indicator, and *CD36* as a good prognostic indicator), though we also found some differences. We also identified a few gene sets that could potentially predict a better response to a specific targeted small molecule inhibitor. These include increased *CREBBP* and *CDKN1A* for KPT-9274, increased *TLR3* for TAK-981, and increased *PI3Kδ*, *AKT3*, and *PTEN*, and decreased *NRAS* for RV1001. The NanoString nCounter® Canine IO panel could be used in future studies to assess these genes in a larger group of dogs using the same combinations of drugs.

## Materials and methods

### Trial design & inclusion criteria

This study was approved by the Tufts University Institutional Animal Care and Use Committee (IACUC), protocol #G2017-110. Hospital-level approval and oversight of this study was done through CSRC approval at Cummings School. Owners of participant dogs provided written, signed informed consent to participate in this study. Documentation throughout the study was completed using RedCap. Animals were not sacrificed for this study. Lymph node aspiration is a standard clinical procedure performed in dogs without anesthesia or analgesia. Dogs were provided with analgesics as necessary throughout the study to alleviate suffering as needed at the discretion of the attending clinician.

This study was part of a larger prospective, non-randomized clinical trial evaluating combination anti-CD20 antibody, doxorubicin, and targeted small molecule inhibitors (RV1001, KPT-9274, TAK-981) in dogs with DLBCL. These combinations were chosen based on activity in DLBCL; KPT-9274 is a dual inhibitor of PAK4 (p21-activated kinase 4) and NAMPT; PAK4 is a serine/threonine protein kinase that promotes tumor cell survival and proliferation, and NAMPT is a critical enzyme for NAD+ salvage pathway synthesis [52, 53]. TAK-981 is a SUMO-activating enzyme inhibitor and induces genes associated with Type I interferon responses [20]. RV1001 is a PI3Kδ inhibitor, a pathway known to be enriched in human and canine DLBCL [11]. Enrollment criteria included a diagnosis of CD21 lymphocytosis/ B cell lymphoproliferative disease on flow cytometry and confirmed as B cell lymphoma on lymph node biopsy, age of at least 1 year, body weight of 10kg or higher, and at least 2 peripheral lymph nodes that measured ≥ 2cm in diameter and adequate organ function as indicated by standard laboratory tests (complete blood count, serum biochemistry profile, urinalysis). Exclusion criteria included pregnant or lactating dogs, evidence of central nervous system involvement, dogs with uncontrolled autoimmune hemolytic anemia or immune mediated thrombocytopenia, dogs with significant cardiovascular disease, dogs that were less than 2 weeks from a major surgical procedure, or dogs that were concurrently on medications that may confound the interpretation of toxicities and/or antitumor activity of the study therapy (including steroid use for more than 48 hours prior to the start of treatment).

All enrolled dogs received anti-CD20 antibody and doxorubicin, in addition to a small molecule inhibitor defined by the study cohort. At the time of publication there have been a total of six arms in this trial; the lymph nodes aspirates from patients in arms 2–4 were analyzed in this study, and their drug protocols are listed below in Table 2.

**Table 2:** Treatment protocols for arms in current study.

Arm	Treatment Protocol
KPT-9274	Anti-CD20 20mg/kg IV q3 weeks for 4 doses
	Doxorubicin 25mg/m2 IV q3 weeks for 4 doses
	KPT-9274 4mg/kg PO q3 weeks for 4 doses
TAK-981	Anti-CD20 10-20mg/kg IV q1 week for 4 doses
	Doxorubicin 25mg/m2 IV once on day 0
	TAK-981 2.5-3mg/kg IV q1 week for 4 doses
RV1001	Anti-CD20 10-20mg/kg IV q3 weeks for 4 doses
	Doxorubicin 20-25mg/m2 IV q3 weeks for 4 doses
	RV1001 10mg/kg PO q24h 4 days on, 3 days off, weekly (12 weeks total or until disease progression)

Anti-CD20 antibody (Elanco) was supplied as a 30.7mg/ml solution and stored at 4°C. Antibody was then diluted to 4mg/ml in 0.9% NaCl for administration. Anti-CD20 antibody was administered at 10-20mg/kg over 90 minutes. KPT-9274 (Karyopharm Therapeutics) was supplied as whole tablets of 5mg, 20mg, and 50mg active ingredient, using pharmaceutical grade excipients. TAK-981 (Takeda Pharmaceutical) was supplied as a 10mg/ml solution and stored at -20°C until IV administration over 30 minutes. RV1001 (Rhizen pharmaceutical) was supplied in gelatin capsules of 25 mg, 100 mg, 250mg, and 400mg active ingredient using pharmaceutical grade excipients. Doxorubicin (Pfizer) was supplied as a 2mg/ml solution.

Lymph node fine needle aspirates were collected from all dogs at baseline (before initiation of therapy), and at time of lymphoma progression as determined by RECIST criteria [54]. In accordance with these criteria, via palpation of peripheral lymph nodes, complete response was considered a return to normal size, partial response was a ≥30% decrease in size, and progressive disease was a ≥20% increase in size.

### RNA isolation & NanoString analysis

RNA from lymph node fine needle aspirates was isolated using Qiashredder columns and Qiagen RNeasy mini kits per the manufacturer’s protocol (both Qiagen). RNA was quantified using a Nanodrop spectrophotometer and 70 ng RNA was used for each hybridization with the nCounter® Canine IO Panel. Hybridization was performed for 19.5h and samples were run on the NanoString nCounter® Sprint instruments per manufacturer instructions.

### ROSALIND® Nanostring gene expression methods

Data was analyzed by ROSALIND® (https://rosalind.bio/), with a HyperScale architecture developed by ROSALIND, Inc. (San Diego, CA). Read Distribution percentages, violin plots, identity heatmaps, and sample MDS plots were generated as part of the QC step. Normalization, fold changes and p-values were calculated using criteria provided by Nanostring. ROSALIND® follows the nCounter® Advanced Analysis protocol of dividing counts within a lane by the geometric mean of the normalizer probes from the same lane. Housekeeping probes to be used for normalization are selected based on the geNorm algorithm as implemented in the NormqPCR R library [55]. Fold changes and p values are calculated using the fast method as described in the nCounter® Advanced Analysis 2.0 User Manual. Criteria for significant genes were chosen as log2fold change less than or greater than 1.5, and padj<0.01 when comparing PD vs baseline, then for individual arms either raw p<0.05 or p<0.01 depending on the group being analyzed. P-value adjustment is performed using the Benjamini-Hochberg method of estimating false discovery rates (FDR). Clustering of genes for the final heatmap of differentially expressed genes was done using the PAM (Partitioning Around Medoids) method using the fpc R library [56] that takes into consideration the direction and type of all signals on a pathway, the position, role and type of every gene, etc. Hypergeometric distribution was used to analyze the enrichment of pathways, gene ontology, domain structure, and other ontologies. The topGO R library [57], was used to determine local similarities and dependencies between GO terms in order to perform Elim pruning correction. Several database sources were referenced for enrichment analysis, including Interpro [58], NCBI [59], MSigDB [60, 61], REACTOME [62], WikiPathways [63]. Enrichment was calculated relative to a set of background genes relevant for the experiment.

### Realtime PCR

RNA was isolated from canine lymph node aspirates using RNeasy kits (Qiagen) per the manufacturer’s protocol. cDNA was prepared using iScript kits (BioRad), and qPCR was performed with SYBR green kits (BioRad) in a CFX96 (BioRad) for 40 cycles at 56°C. Copy number was calculated using ΔΔCT in Microsoft Excel.

### Statistical analysis

Statistical analysis for NanoString and Rosalind analysis were performed as described above. Comparison of individual gene expression for both nCounter and PCR between poor responders and good responders was performed with unpaired t-tests in Prism Graphpad, with significant genes determined as p<0.05. TTP and OST were determined using the Kaplan-meier product limit method, and significant was calculated using Cox proportional hazards, with significance set at p < 0.05.

## Supporting information

S1 File (XLSM)

S2 File (XLSX)

S1 Graphical abstract (DOCX)
