# Supplementary material for: Baseline tumor gene expression signatures correlate with chemoimmunotherapy treatment responsiveness in canine B cell lymphoma
Source: PLoS One. 2023 Aug 25;18(8):e0290428. doi: 10.1371/journal.pone.0290428 (PMC10456153; doi:10.1371/journal.pone.0290428)
Supplement: S1 Graphical abstract — (DOCX) [file pone.0290428.s003.docx]

**Graphical abstract**.


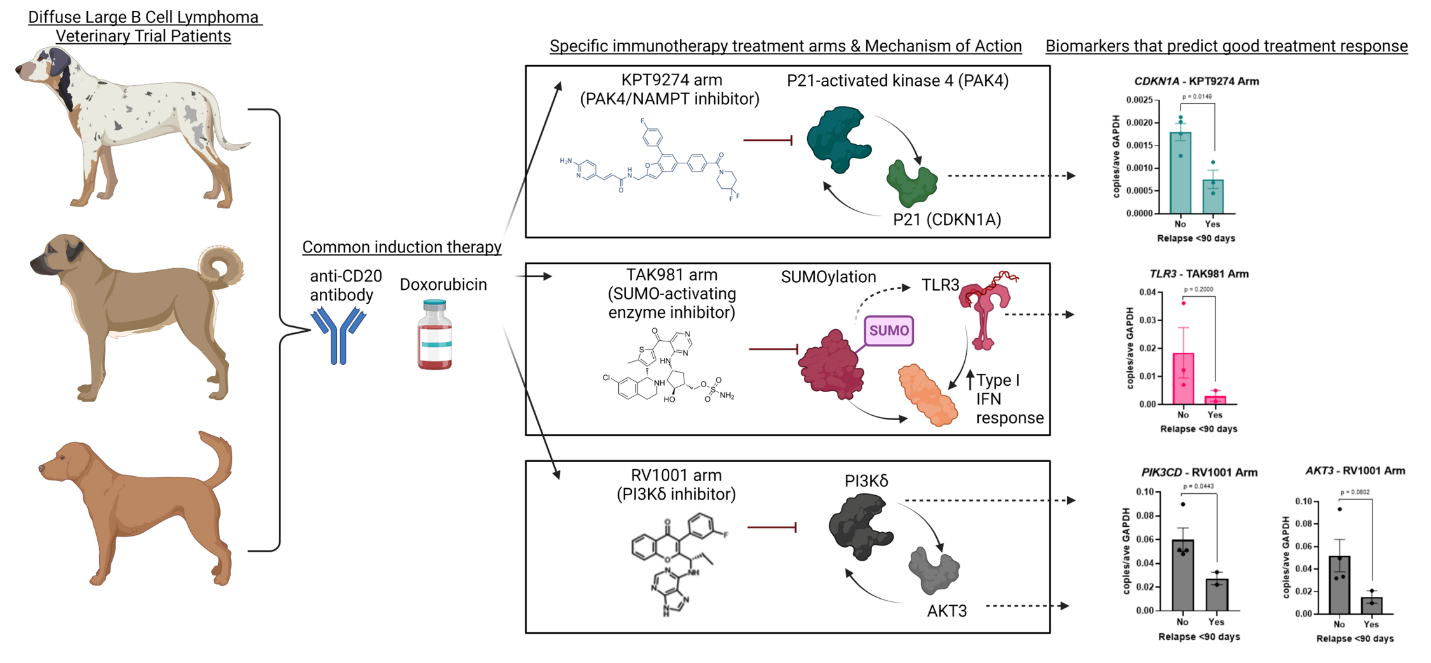


Veterinary trial for canine diffuse large B cell lymphoma was conducted using standard induction therapy of anti-CD20 antibody and doxorubicin, followed by randomization into 3 different immunotherapy trial arms as indicated. Biomarker genes from baseline tumors helped predict which dogs would do well versus those who did poorly as defined by relapse greater than or less than 90 days, respectively. These biomarker genes tended to correspond to the drug mechanism of action.
